# Contribution of Aerobic Physiological Variables to 500 m Sprint Kayak Performance in National-Level Athletes: A Hierarchical Regression Analysis

**DOI:** 10.3390/sports14080363

**Published:** 2026-08-20

**Authors:** Apostolos Papandreou, Giorgos Paradisis, Anastassios Philippou, Elias Zacharogiannis

**Affiliations:** 1 School of Physical Education and Sport Science, National and Kapodistrian University of Athens, 17237 Athens, Greece; gparadi@phed.uoa.gr (G.P.); elzach@phed.uoa.gr (E.Z.); 2 Department of Physiology, Medical School, National and Kapodistrian University of Athens, 11527 Athens, Greece; tfilipou@med.uoa.gr

**Keywords:** sprint kayaking, physiological determinants, power output, ventilatory threshold, power output at 75% of maximal oxygen uptake, aquatic sports

## Abstract

The purpose of this study was to examine the independent contribution of aerobic physiological variables to 500 m sprint kayak performance in national-level male athletes. Twenty-four male flatwater kayak athletes completed post-intervention physiological and performance assessments following a 6-week training intervention. Sprint performance was assessed using a 500 m kayak ergometer time trial. Pearson correlation and hierarchical multiple regression analyses were performed after accounting for training-group allocation. Power output at VO_2_max (POVO_2_max) demonstrated the strongest association with 500 m performance time (r = −0.71, *p* < 0.001). Hierarchical multiple regression analysis showed that training-group allocation explained a small, non-significant proportion of the variance in performance time (R^2^ = 0.082, *p* = 0.648). The addition of POVO_2_max significantly increased the explained variance (R^2^ = 0.708, ΔR^2^ = 0.626, *p* < 0.001); whereas, VO_2_max (ΔR^2^ = 0.012, *p* = 0.363) and post-exercise blood lactate concentration (ΔR^2^ = 0.004, *p* = 0.855) did not provide additional explanatory value. These findings indicate that POVO_2_max was the strongest independent physiological predictor of 500 m sprint kayak performance after accounting for training-group allocation.

## 1. Introduction

Sprint kayaking performance in the Olympic 500 m event requires the integration of aerobic and anaerobic energy production, neuromuscular coordination, and sport-specific technical efficiency [1,2,3,4]. Given the race duration (~100–130 s), athletes must generate and sustain greater levels of mechanical power while tolerating substantial metabolic stress [2,3,5]. Both oxidative and glycolytic energy systems contribute significantly to performance. During a 500 m sprint kayak race, oxidative metabolism is estimated to provide approximately 60–70% of the total energy demand; whereas, anaerobic pathways contribute the remaining 30–40%, particularly during the start and final sprint. Consequently, success in sprint kayaking appears to depend largely on the ability to sustain greater functional power output throughout the race [1,6,7]. Maximal oxygen uptake (VO_2_max) has traditionally been considered an important determinant of endurance performance [1,6,8]. Nevertheless, in national-level athletes, VO_2_max often demonstrates limited sensitivity for distinguishing performance capacity because aerobic fitness becomes relatively homogeneous [6,8,9]. Furthermore, previous research has shown that VO_2_max values measured during kayak-specific ergometer testing are approximately 20% less than those typically reported in endurance sports involving a larger active muscle mass. This difference is primarily attributed to the relatively smaller amount of muscle mass engaged during upper-body dominant paddling exercise, which limits maximal oxygen extraction despite substantial cardiovascular demand [10,11,12]. Consequently, increasing attention has been directed toward functional physiological variables, particularly power output at VO_2_max (POVO_2_max), which reflects the athlete’s ability to translate aerobic capacity into external mechanical power during sport-specific exercise [2,8,10,11]. Additional physiological characteristics, including power output at the second ventilatory threshold (POVT_2_), power output at 75% of maximal oxygen uptake (PO_75_%VO_2_max), blood lactate concentration, and maximal heart rate (HRmax), may also influence sprint kayak performance by reflecting metabolic tolerance, movement efficiency, and cardiovascular responses to high-intensity exercise [4,5,10,11,12,13]. In paddle sports, where propulsion is directly dependent on force application and stroke efficiency, these variables may provide complementary information regarding the interaction between physiological capacity and mechanical power production. The second ventilatory threshold represents an important physiological marker associated with increased metabolic acidosis and reduced exercise tolerance during high-intensity exercise [10,13]. Similarly, paddling economy reflects the efficiency with which metabolic energy is converted into external mechanical work and may influence the ability to sustain race pace during middle-duration sprint events [6,8]. Blood lactate concentration has also been widely used as an indicator of the metabolic response to maximal exercise, although its independent contribution to sprint kayak performance remains unclear [2,14,15,16]. Previous studies have demonstrated associations between aerobic power, threshold-related physiological variables, and kayaking performance [2,14,15,16]. Moreover, recent investigations in trained sprint kayakers have highlighted the importance of peripheral adaptations, muscle oxygenation, and sport-specific aerobic power characteristics for performance development [16]. High-intensity interval training (HIIT) and sprint interval training (SIT) have also been shown to induce significant improvements in aerobic fitness, physiological adaptations, and performance-related variables in endurance and paddle sport athletes [17,18,19,20,21,22,23,24]. Despite the growing body of literature examining physiological responses to interval training and sprint kayaking performance, limited evidence exists regarding the independent contribution of multiple physiological variables to 500 m sprint kayaking performance using multivariable predictive approaches [2,14,16]. Because physiological variables are closely interrelated in trained athletes, simple correlation analyses cannot determine their independent contribution to performance [6,8,9]. Furthermore, physiological characteristics measured following a training intervention may still be influenced by the intervention itself. Therefore, statistical models that account for potential training-group effects provide a more robust evaluation of independent physiological predictors. Hierarchical multiple regression is particularly suitable for this purpose because it allows potential confounding variables, such as training-group allocation, to be controlled before evaluating the additional contribution of physiologically meaningful predictors [25,26,27,28,29]. Therefore, the purpose of the present study was to examine the relationship between post-intervention physiological variables and laboratory 500 m sprint kayak performance assessed on a kayak ergometer in national-level male athletes. A secondary aim was to determine the independent contribution of selected physiological variables to sprint performance using correlation analyses and hierarchical multiple regression, while accounting for training-group allocation before evaluating the incremental contribution of power output at VO_2_max, maximal oxygen uptake, and blood lactate concentration [25,26,27,28,29]. It was hypothesized that POVO_2_max would emerge as the strongest independent predictor of 500 m sprint kayak performance; whereas, VO_2_max and blood lactate concentration would provide little additional predictive value after adjustment for training-group allocation.

## 2. Materials and Methods

### 2.1. Participants

Twenty-four male national-level flatwater kayak athletes participated in the present study. All participants had competed in official national flatwater kayak competitions for at least four years and were free from injury or illness at the time of testing [5,9,14,15,17]. The present investigation represents a secondary performance-prediction analysis derived from a larger randomized training intervention. Participants were originally recruited through an open invitation published on the official website of the Hellenic Canoe–Kayak Federation. To minimize sex-related physiological variability and improve the homogeneity of the prediction model, only male athletes were included in the present analyses. The participants had a mean (±SD) age of 18.6 ± 4.21 years, height of 176.09 ± 8.76 cm, and body mass of 73.09 ± 11.03 kg. Before the original intervention, participants were matched according to sex and baseline 500 m kayak ergometer performance and subsequently allocated to one of four intervention groups (HIIT, SIT, COMB, or CON; *n* = 6 per group) using a matched randomized design. During the intervention period, only low-intensity recreational activities (e.g., cycling, swimming, and recreational sport games) were permitted outside the prescribed training program. Participants were excluded if they sustained an injury or illness preventing completion of the physiological assessments, used medications or substances known to influence physiological responses, or failed to complete all physiological and performance testing sessions. All athletes were familiarized with the testing procedures and kayak ergometer during a one-week familiarization period before baseline testing [13,27]. All laboratory assessments were conducted under standardized environmental conditions (20–21 °C, 50–55% relative humidity) between 12:00 and 16:00 h [13,14]. Participants refrained from strenuous exercise for 24 h before testing and consumed a standardized light meal approximately 3 h before each assessment [11,17]. Written informed consent was obtained from all participants and, when applicable, from their legal guardians. The study was approved by the Ethics Committee of the National and Kapodistrian University of Athens (Protocol No. 3422/Δ5, 12 January 2022), and all procedures conformed to the principles of the Declaration of Helsinki [30].

### 2.2. Study Design

The present study represents a secondary observational analysis derived from a larger randomized controlled training intervention conducted in national-level flatwater kayak athletes. The original intervention was designed to compare the physiological adaptations induced by different training modalities; whereas, the objective of the present study was to identify the physiological determinants of 500 m sprint kayak performance following completion of the intervention. To improve the homogeneity of the prediction model and minimize biological variability associated with sex-related physiological differences, the present analyses were restricted to male athletes. Accordingly, only post-intervention (POST) physiological and performance data obtained from the 24 male participants were included in the predictive analyses. The primary purpose of the present investigation was not to evaluate training-induced adaptations from PRE to POST, but to determine which laboratory-derived physiological variables independently explained 500 m sprint kayak performance after completion of the intervention. Because all participants completed an identical standardized detraining period before baseline testing, POST measurements were considered to best represent the athletes’ physiological status at the time of performance assessment. This analytical approach was selected because the objective of the present study differed from that of the original randomized intervention. Whereas the primary intervention study examined longitudinal physiological adaptations from PRE to POST, the present investigation aimed to identify the physiological determinants of 500 m sprint kayak performance within the specific physiological state achieved following completion of the intervention. Consequently, PRE measurements were intentionally not included in the regression analyses. This decision was based on three methodological considerations. First, the standardized detraining period preceding baseline testing meant that PRE measurements reflected a detrained physiological state rather than the condition in which performance prediction was examined. Second, although participants were allocated using a matched randomized design, individual baseline physiological variability remained, and inclusion of PRE values would have addressed a different research question concerning longitudinal physiological adaptations rather than cross-sectional performance prediction. Finally, the present regression models were developed to identify physiological variables associated with performance following completion of the intervention and should therefore not be interpreted as a general predictive model applicable to athletes under different training conditions or competitive phases, but rather as models describing performance within this specific post-intervention physiological state.

**Figure 1:**
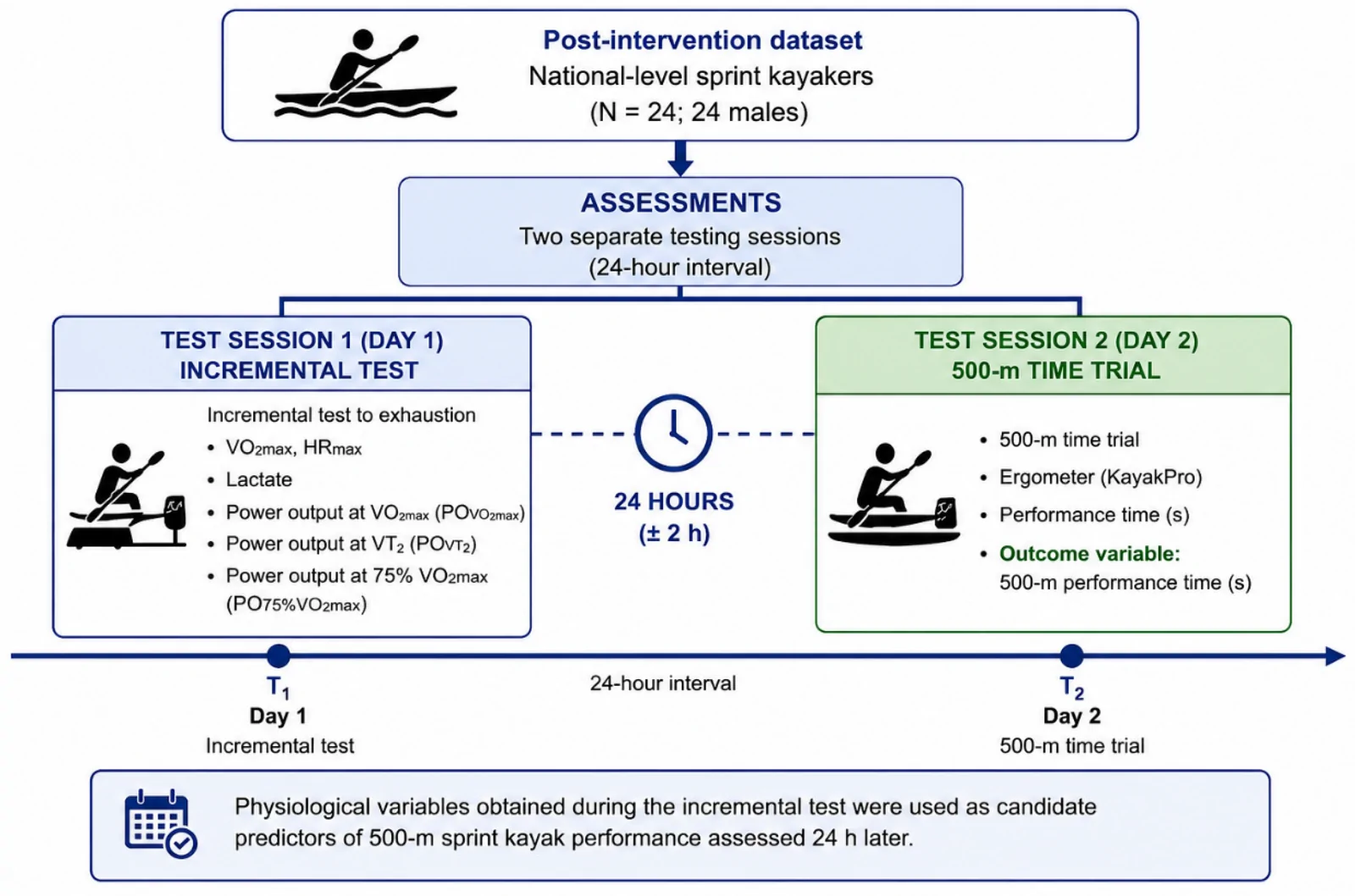
Testing design and assessment timeline. Following completion of the 6-week training intervention, twenty-four male national-level sprint kayak athletes completed two standardized laboratory testing sessions separated by 24 h. Physiological variables were assessed during an incremental kayak ergometer test (Day 1), followed by a 500 m kayak ergometer time trial (Day 2). Laboratory-derived physiological variables were subsequently entered into hierarchical regression models to identify independent predictors of 500 m sprint performance after adjustment for intervention group.

Participants completed a 6-week supervised training intervention consisting of three training sessions per week under the supervision of experienced canoe–kayak coaches. Although athletes originated from four different intervention groups (high-intensity interval training [HIIT], sprint interval training [SIT], combined training [COMB], and control [CON]), the intervention itself was not the primary focus of the present investigation. Instead, intervention group was entered as a control variable in the hierarchical regression analyses to account for potential residual effects of the different training programs while evaluating the independent contribution of physiological variables to sprint performance. The testing design and assessment timeline are presented in Figure 1.

### 2.3. Incremental Physiological Testing

Participants performed an incremental exercise test to volitional exhaustion on a kayak ergometer (KayakPro Compact, KayakPro LLC, Miami Beach, FL, USA) [31]. Ergometer settings, including seat position, footrest configuration, and resistance level, were standardized and replicated during all testing sessions [13,31]. Resistance level was set at level 7 for all participants. Before the incremental test, participants completed a standardized 5 min warm-up at approximately 70% of VO_2_max [8,11]. The incremental protocol commenced at 50 W, with workload increasing by 20 W every 2 min until volitional exhaustion. Total exercise duration ranged between 10 and 14 min [32]. Pulmonary gas exchange variables were measured continuously throughout the test using a calibrated metabolic cart system (MedGraphics, Medical Graphics Corporation, St. Paul, MN, USA) with breath-by-breath analysis and 8–10 breath averaging [13,33]. Flow calibration was performed using a 3 L syringe, and gas analyzers were calibrated before each test using standardized reference gases [33]. Maximal oxygen uptake (VO_2_max) was determined when at least two of the following criteria were achieved: (a) a plateau in VO_2_ despite increased workload, (b) respiratory exchange ratio (RER) ≥ 1.15, (c) heart rate ≥ 90% of age-predicted maximum, and/or (d) volitional exhaustion [32,33]. Heart rate was continuously recorded throughout the protocol using telemetry (Polar Electro Oy, Kempele, Finland) [34,35].

### 2.4. Physiological Variables

Power output at maximal oxygen uptake (POVO_2_max) was defined as the maximal power output attained during the incremental exercise test [2,6]. Second ventilatory threshold (POVT_2_) was identified using established ventilatory criteria, including disproportionate increases in ventilation relative to carbon dioxide production, concurrent increases in the ventilatory equivalents for oxygen (VE/VO_2_) and carbon dioxide (VE/VCO_2_), reductions in end-tidal carbon dioxide pressure (PETCO_2_), and increases in end-tidal oxygen pressure (PETO_2_), according to the recommendations of Karlman Wasserman and colleagues [10,33]. Threshold determination was performed independently by two experienced investigators. In cases of disagreement, a third investigator reviewed the data, and a consensus decision was reached. Inter-rater reliability for ventilatory threshold determination was excellent (ICC > 0.90). The power output corresponding to VT_2_ represented the workload at which the second ventilatory threshold occurred and should not be interpreted as the maximal workload achieved during the incremental exercise test. Following VT_2_ determination, all participants continued the protocol until volitional exhaustion according to the predefined termination criteria. Capillary blood lactate concentration was determined from fingertip blood samples collected 3 min after completion of the incremental test using an enzymatic–amperometric analyzer [10]. Maximal heart rate (HRmax) was defined as the maximal heart rate attained during the incremental protocol [34,35]. Power output at 75% VO_2_max (PO_75_%VO_2_max) was operationally defined as the external mechanical power output corresponding to 75% of each participant’s maximal oxygen uptake (VO_2_max) during the incremental kayak ergometer test. For each athlete, 75% of the individual VO_2_max value was first calculated, and the corresponding mechanical power output was subsequently identified from the breath-by-breath gas-exchange and workload data obtained during the incremental test. Higher PO_75_%VO_2_max values indicated that a greater external power output was produced at the same relative oxygen uptake. The exercise intensity corresponding to 75% of VO_2_max was selected because it represents a stable submaximal physiological domain in which oxygen uptake generally reaches a steady state while remaining sufficiently demanding to reflect sport-specific aerobic efficiency and sustainable mechanical power production [19,36]. In a previous study involving national-level kayak athletes, paddling speed at 75% of VO_2_max was used as a sport-specific indicator of paddling economy and was shown to be sensitive to training-induced adaptations [36]. Although the present study used power output rather than paddling speed at the same relative exercise intensity, both variables are derived from the same physiological construct and represent complementary sport-specific indices of submaximal aerobic performance. Consequently, differences in external mechanical power output achieved at the same relative oxygen uptake may provide an indirect indicator of paddling efficiency during prolonged submaximal exercise.

The corresponding workload was calculated individually for each athlete based on the power output attained at VO_2_max during the incremental exercise test and was used as an index of submaximal paddling efficiency [2,3,25]. Because the primary objective of the present study was to determine whether sport-specific maximal aerobic power provides incremental predictive value beyond conventional physiological indices, the hierarchical regression model was specified as a priori. Accordingly, POVO_2_max was selected as the primary functional predictor; whereas, VO_2_max and post-exercise blood lactate concentration were subsequently entered to evaluate their additional contribution to 500 m sprint kayak performance. Although POVT_2_ and PO_75_%VO_2_max were also assessed, they were not included in the hierarchical model because they represent related sport-specific power variables reflecting similar physiological characteristics to POVO_2_max [2,6,14,16,25]. Including both variables in the same hierarchical regression model would have introduced conceptual redundancy and increased the potential for multicollinearity because both represent closely related sport-specific power indices derived from the same incremental exercise test. Therefore, the hierarchical regression model was designed to evaluate the incremental predictive value of maximal sport-specific aerobic power (POVO_2_max) beyond conventional physiological variables rather than to compare highly correlated sport-specific power measures.

### 2.5. 500 m Sprint Performance Test

Sprint kayak performance was assessed using a simulated 500 m time trial performed on a kayak ergometer (KayakPro Compact, KayakPro LLC, Miami Beach, FL, USA) under standardized laboratory conditions [3,5,27]. Following a standardized warm-up, participants completed the trial at maximal effort, and total performance time (s) was recorded. Athletes were instructed to complete the 500 m distance as quickly as possible while adopting their preferred pacing strategy throughout the trial [5,32,33]. Ergometer settings, including seat position, footrest configuration, and resistance level, were identical to those used during the incremental physiological assessment to ensure consistency between testing sessions [13,31].

### 2.6. Statistical Analysis

Data are presented as mean ± standard deviation (SD). Normality and homogeneity of variance were assessed using the Shapiro–Wilk and Levene’s tests, respectively. Pearson product–moment correlation coefficients were calculated to examine the relationships between post-intervention physiological variables and 500 m sprint kayak performance time [25]. Correlation coefficients were interpreted as trivial (<0.10), small (0.10–0.29), moderate (0.30–0.49), large (0.50–0.69), very large (0.70–0.89), and nearly perfect (≥0.90) [25,37]. Negative correlation coefficients indicated that higher physiological values were associated with shorter (faster) 500 m performance times. Hierarchical multiple linear regression analysis was performed to determine the independent contribution of selected physiological variables to 500 m sprint kayak performance after accounting for training-group allocation [38]. Sprint performance time (500 m) was specified as the dependent variable. Training groups (HIIT, SIT, COMB, and CON) were entered in the first block as a control variable to account for potential residual effects of the intervention. Power output at maximal oxygen uptake (POVO_2_max) was entered in the second block because it represented the primary physiological variable of interest and demonstrated the strongest association with sprint performance. Maximal oxygen uptake (VO_2_max) was subsequently entered in the third block to determine whether maximal aerobic capacity provided additional explanatory value beyond sport-specific functional aerobic power. Post-exercise blood lactate concentration was entered in the fourth block to evaluate whether the metabolic response to maximal exercise explained additional variance in sprint performance beyond that accounted for by the preceding variables. For each regression model, standardized regression coefficients (β), unstandardized regression coefficients (B), standard errors (SE), 95% confidence intervals (95% CI), coefficients of determination (R^2^), adjusted coefficients of determination (adjusted R^2^), changes in explained variance (ΔR^2^), F-change statistics, and corresponding *p* values were calculated. Regression assumptions were evaluated before interpretation of the models. Multicollinearity was assessed using tolerance and variance inflation factor (VIF), with VIF values < 5 considered acceptable. Independence of residuals was evaluated using the Durbin–Watson statistic, while influential observations were assessed using Cook’s distance, leverage values, and studentized residuals. Visual inspection of standardized residual plots and normal probability (Q–Q) plots was performed to verify linearity, homoscedasticity, and normality of residuals. Inter-rater reliability for ventilatory threshold determination was assessed using intraclass correlation coefficients (ICC), with values < 0.50 considered poor, 0.50–0.74 moderate, 0.75–0.89 good, and ≥0.90 excellent reliability [25]. Statistical significance was accepted at *p* < 0.05. The order of predictor entry was determined a priori based on the study hypothesis and physiological rationale. All statistical analyses were performed using IBM SPSS Statistics (Version 29.0; IBM Corp., Armonk, NY, USA).

## 3. Results

### 3.1. Descriptive Statistics

Table 1 presents the descriptive statistics for the post-intervention physiological and performance variables obtained from the 24 male national-level sprint kayak athletes. Mean VO_2_max was 45.24 ± 7.84 mL·kg^−1^·min^−1^; whereas, mean POVO_2_max and POVT_2_ were 102.80 ± 25.88 W and 80.83 ± 19.22 W, respectively. Mean Power output at 75% VO_2_max (PO_75_%VO_2_max) was 76.79 ± 19.83 W. Blood lactate concentration measured 3 min after the incremental exercise test was 10.38 ± 2.75 mmol·L^−1^, while mean HRmax was 196.79 ± 6.52 beats·min^−1^. Mean 500 m sprint performance time was 127.21 ± 6.07 s.

### 3.2. Correlation Analysis

**Figure 2:**
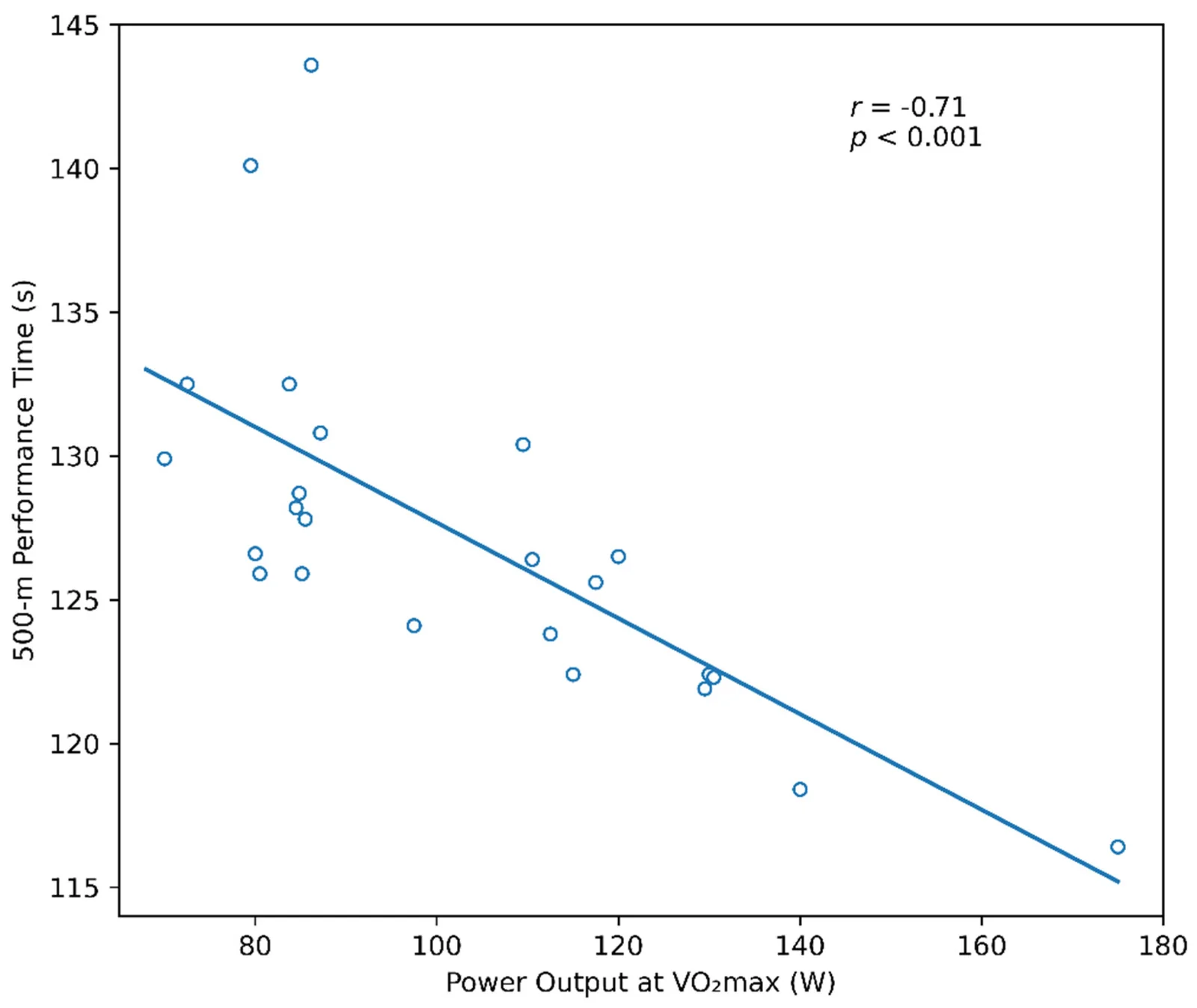
Relationship between post-intervention power output at maximal oxygen uptake (POVO_2_max) and 500 m sprint kayak performance time in national-level athletes. Each point represents an individual participant. The regression line represents the linear association with the corresponding 95% confidence interval.

Pearson product–moment correlation coefficients between post-intervention physiological variables and 500 m sprint performance time are presented in Table 2. POVO_2_max demonstrated the strongest negative association with sprint performance time (r = −0.71, *p* < 0.001), indicating that athletes with greater maximal aerobic power achieved faster 500 m sprint performances. Significant negative correlations were also observed for POVT_2_ (r = −0.54, *p* = 0.006) and PO_75_%VO_2_max (r = −0.69, *p* < 0.001). In contrast, VO_2_max, HRmax, and blood lactate concentration were not significantly associated with sprint performance time (*p* > 0.05). The relationship between post-intervention POVO_2_max and 500 m sprint performance time is illustrated in Figure 2.

### 3.3. Hierarchical Multiple Regression Analysis

**Figure 3:**
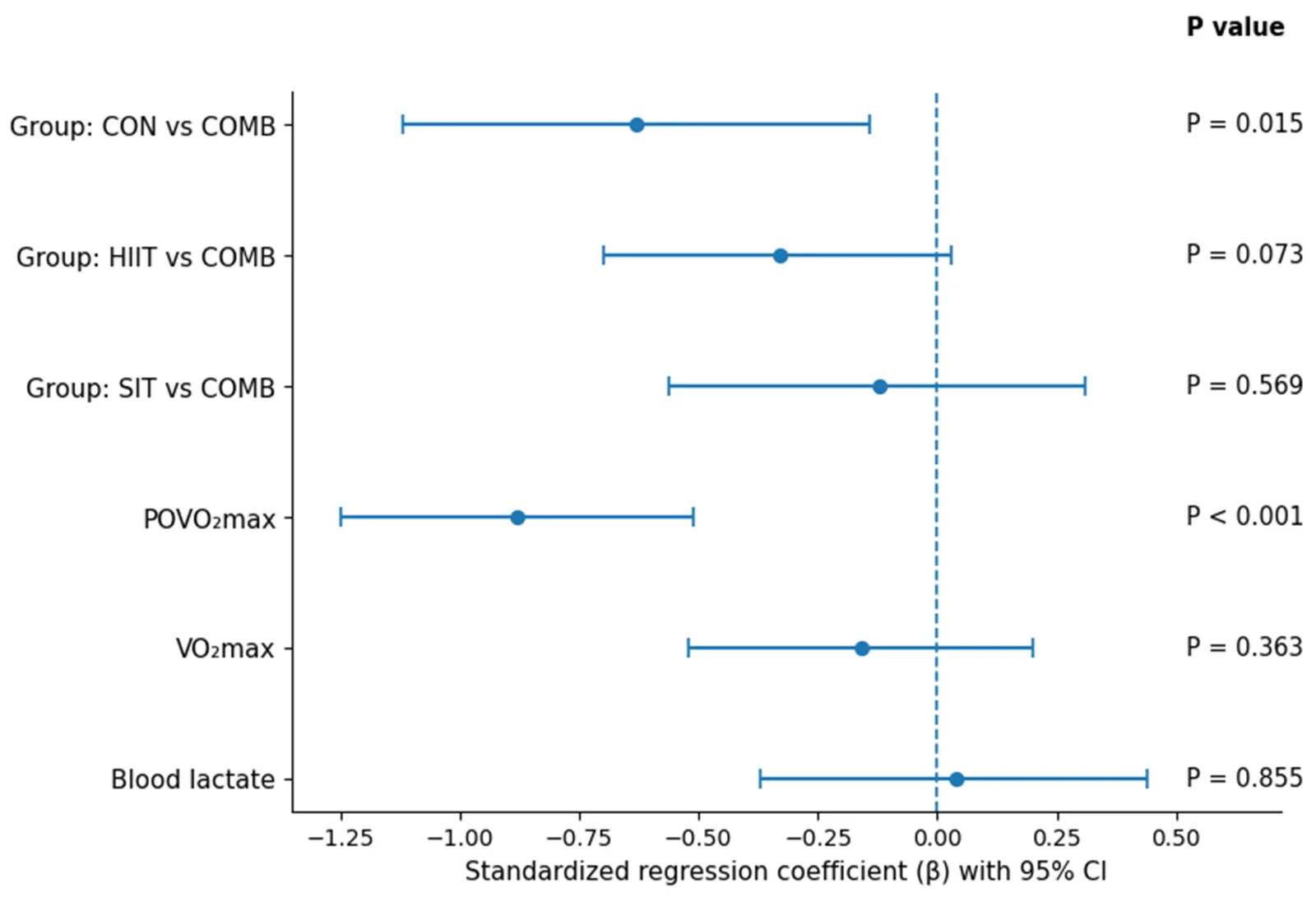
Forest plot showing regression coefficients and corresponding 95% confidence intervals from the final hierarchical multiple regression model predicting 500 m sprint kayak performance in male athletes (*N* = 24). Negative coefficients indicate that higher predictor values were associated with faster (lower) 500 m performance times. Among the examined physiological variables, only power output at maximal oxygen uptake (POVO_2_max) remained an independent predictor of performance after adjustment for intervention group, maximal oxygen uptake (VO_2_max), and blood lactate concentration.

Hierarchical multiple regression analysis was performed to determine whether selected physiological variables explained variance in 500 m sprint kayak performance time beyond that attributable to training-group allocation. The results of the hierarchical regression analyses, including the standardized regression coefficients, changes in explained variance (ΔR^2^), and model statistics, are presented in Table 3 and Table 4. In the first model, training group accounted for a small and non-significant proportion of the variance in performance time (R^2^ = 0.082, *p* = 0.648). The addition of POVO_2_max in the second model significantly improved the model, increasing the explained variance to 70.8% (R^2^ = 0.708, ΔR^2^ = 0.626, *p* < 0.001), indicating that POVO_2_max was the strongest independent predictor of 500 m sprint kayak performance. The addition of VO_2_max in Model 3 produced only a small and non-significant increase in explained variance (ΔR^2^ = 0.012, *p* = 0.363). The addition of post-exercise blood lactate concentration in Model 4 resulted in a further trivial and non-significant increase in explained variance (ΔR^2^ = 0.004, *p* = 0.855). Standardized regression coefficients and corresponding 95% confidence intervals are presented in Figure 3. Negative standardized beta coefficients indicated that greater physiological values were associated with faster 500 m sprint kayak performance.

Final hierarchical multiple regression equation

Predicted 500 m Time (s) = 157.31 − 0.122(VO_2_max) − 0.206(POVO_2_max) + 0.078(La^+2^) − 8.67(CON) − 4.53(HIIT) − 1.65(SIT).

## 4. Discussion

Despite the recognized importance of aerobic physiological characteristics for sprint kayak performance, limited evidence is available regarding the independent contribution of sport-specific physiological variables to 500 m performance following a standardized training intervention [1,2,3,4,5,6]. Previous studies have primarily examined simple associations between physiological variables and performance or have focused on training-induced adaptations [2,3,6,14,16]; whereas, few have evaluated the relative contribution of these variables within a multivariable predictive framework [14,16]. Addressing this knowledge gap may improve the interpretation of laboratory-based physiological assessments and assist coaches in identifying the physiological characteristics most closely associated with sprint kayak performance [2,6,14]. Therefore, the purpose of the present study was to examine the relationship between post-intervention physiological variables and 500 m sprint kayak performance and to determine their independent contribution using hierarchical multiple regression analysis. The principal finding was that power output at VO_2_max remained the strongest independent predictor of sprint kayak performance after adjustment for intervention group; whereas, VO_2_max and post-exercise blood lactate concentration did not provide additional explanatory value.

The purpose of the present study was to examine the contribution of aerobic physiological variables to 500 m sprint kayak performance in national-level male athletes using correlational and hierarchical regression analyses. The primary finding was that power output at maximal oxygen uptake (POVO_2_max) was the strongest independent physiological predictor of sprint performance after accounting for training-group allocation. Although training-group allocation alone explained only a small and non-significant proportion of the variance in 500 m sprint performance, the addition of POVO_2_max markedly improved the regression model. In contrast, the subsequent inclusion of VO_2_max and post-exercise blood lactate concentration did not significantly improve the model, indicating that these variables provided no additional predictive information beyond that explained by POVO_2_max. The final hierarchical regression model explained 72.4% of the variance in 500 m sprint kayak performance, with POVO_2_max emerging as the strongest independent physiological predictor in the present cohort. The present analysis should be interpreted as a post-intervention predictor study rather than an evaluation of training-induced adaptations. Although the physiological characteristics were influenced by the preceding intervention, the primary objective was to identify the physiological variables most strongly associated with sprint kayak performance following completion of training. Accordingly, training-group allocation was entered in the first block of the hierarchical regression model to account for potential residual effects of the intervention before evaluating the independent contribution of the physiological variables. The findings support our initial hypothesis that sport-specific power-related physiological characteristics provide greater predictive value than traditional physiological indices. Specifically, POVO_2_max remained the only physiological variable that significantly improved the regression model after adjustment for training-group allocation. In contrast, VO_2_max did not explain additional variance in performance once POVO_2_max was included in the model, while post-exercise blood lactate concentration also failed to provide incremental predictive value. These findings suggest that maximal aerobic capacity alone and the magnitude of post-exercise lactate accumulation were insufficient to independently distinguish sprint kayak performance in trained athletes. The physiological distinction between VO_2_max and POVO_2_max may explain these findings. Whereas VO_2_max reflects maximal aerobic capacity, POVO_2_max represents the external mechanical power an athlete is able to generate while exercising at maximal oxygen uptake. Consequently, POVO_2_max integrates both metabolic capacity and sport-specific mechanical performance, providing a more functionally relevant indicator of sprint kayaking ability. In flatwater sprint kayaking, propulsion depends not only on oxygen delivery but also on the effective conversion of metabolic energy into external paddling power through coordinated force production and technical efficiency [2,5,12,39]. The persistence of POVO_2_max as the only independent physiological predictor therefore suggests that the ability to translate aerobic capacity into external mechanical power was more important for sprint kayak performance than maximal oxygen uptake alone. These findings are consistent with previous investigations demonstrating that sport-specific power variables are more strongly associated with kayak performance than conventional cardiorespiratory indices [2,6,14,16]. In well trained athletes, VO_2_max frequently exhibits limited discriminatory capacity because aerobic fitness becomes relatively homogeneous; whereas, variables reflecting the mechanical expression of aerobic capacity better distinguish competitive performance [6,8,9]. The absence of an independent contribution of post-exercise blood lactate concentration further suggests that metabolic accumulation alone is insufficient to explain inter-individual differences in sprint kayak performance. Despite the relatively substantial explanatory power of the final regression model (R^2^ = 0.72), approximately 28% of the variance in performance remained unexplained. This finding indicates that additional determinants—including neuromuscular characteristics, paddling technique, stroke mechanics, pacing strategy, psychological factors, and environmental conditions—also contribute to sprint kayak performance. Accordingly, sprint kayaking should be considered a multifactorial performance task requiring the interaction of physiological, biomechanical, technical, tactical, and psychological components. From a practical perspective, the present findings support the incorporation of sport-specific power assessments, particularly POVO_2_max, into physiological testing batteries and athlete-monitoring programs. Monitoring the athlete’s ability to generate external power at maximal aerobic capacity may represent a more informative physiological target than focusing exclusively on VO_2_max or post-exercise blood lactate concentration when evaluating competitive sprint kayak athletes.

### 4.1. VO_2_max, Blood Lactate, and Sprint Kayak Performance

Although maximal oxygen uptake (VO_2_max) has traditionally been regarded as a key determinant of endurance performance, the present findings indicate that it did not provide additional predictive value for 500 m sprint kayak performance once power output at VO_2_max (POVO_2_max) had been considered. VO_2_max demonstrated only a moderate, non-significant correlation with sprint performance and did not significantly improve the hierarchical regression model following the inclusion of training-group allocation and POVO_2_max. These findings suggest that maximal aerobic capacity alone is insufficient to explain performance differences among well-trained sprint kayakers and are consistent with previous studies reporting that VO_2_max becomes relatively homogeneous in trained athletes, thereby reducing its ability to discriminate performance level [6,8,9]. In contrast, POVO_2_max represents the external mechanical power generated while exercising at maximal oxygen uptake and therefore integrates aerobic capacity with sport-specific force production and paddling efficiency. Consequently, the ability to translate aerobic metabolism into external mechanical power appears to be more relevant for sprint kayak performance than maximal oxygen uptake itself [2,14,16]. Similarly, post-exercise blood lactate concentration was neither significantly associated with sprint performance nor did it provide additional explanatory value in the hierarchical regression model. Although blood lactate concentration reflects glycolytic activation and metabolic stress during maximal exercise, it represents the net balance between lactate production, buffering, distribution, and clearance rather than the athlete’s capacity to sustain external mechanical power output [10]. Consequently, athletes with similar sprint performances may exhibit substantially different post-exercise lactate concentrations, limiting its usefulness as an independent predictor of competitive performance. Collectively, these findings indicate that sport-specific functional power output provides greater predictive information than conventional physiological indices such as VO_2_max and post-exercise blood lactate concentration. From a practical perspective, physiological assessments that quantify the athlete’s ability to generate external power during maximal aerobic exercise, particularly POVO_2_max, may provide more meaningful information for athlete monitoring than traditional measures of aerobic capacity or metabolic stress.

### 4.2. Practical Applications

From a practical perspective, the findings of the present study emphasize the importance of monitoring sport-specific functional aerobic power during sprint kayak training and performance evaluation [5,9,17]. Coaches and sport scientists may consider incorporating physiological assessments capable of quantifying the athlete’s ability to generate external mechanical power during maximal exercise in addition to traditional measures such as maximal oxygen uptake (VO_2_max) and post-exercise blood lactate concentration [1,6]. Power output at VO_2_max (POVO_2_max) emerged as the strongest independent predictor of 500 m sprint kayak performance after accounting for training-group allocation, highlighting its practical value for athlete monitoring, talent identification, and performance evaluation. In contrast, neither VO_2_max nor post-exercise blood lactate concentration provided additional predictive value once POVO_2_max had been included in the hierarchical regression model. These findings suggest that assessments focusing on sport-specific functional power provide more informative performance-related information than conventional physiological indices alone. Although the present study was not designed to evaluate training interventions, previous studies have shown that high-intensity interval training can improve sport-specific aerobic power and performance-related physiological adaptations in trained athletes [16,18,19,20,21,22,23]. Consequently, routine assessment of POVO_2_max may provide a useful tool for monitoring physiological adaptations and supporting training prescription in competitive sprint kayaking [5,17].

### 4.3. Limitations

Certain limitations of the present study should be acknowledged. First, the predictive analyses were based exclusively on post-intervention (POST) values. Although this approach was consistent with the aim of identifying the physiological determinants of sprint kayak performance following completion of the intervention, residual differences between training groups may have influenced the observed associations. To account for this potential source of variability, training-group allocation was entered as the first block of the hierarchical regression model before the physiological variables were evaluated. Future studies should examine both post-intervention values and training-induced changes (Δ values) to determine their relative contribution to sprint performance. In addition, the POST-only analytical approach adopted in the present study should not be interpreted as providing a universal prediction model applicable across different training phases or athlete populations. Rather, the hierarchical regression models describe the relationships between physiological characteristics and 500 m sprint kayak performance within the specific physiological state achieved following completion of the intervention. Consequently, because PRE measurements were intentionally not incorporated into the regression analyses, the present study cannot determine how longitudinal changes in physiological variables influence subsequent performance improvements or establish causal relationships between training-induced adaptations and sprint performance. Future longitudinal investigations incorporating repeated physiological assessments across different phases of training are warranted to further clarify these relationships.

Second, the analyses were based on a relatively small sample of national-level male athletes. Although the sample size was comparable to previous studies in competitive kayaking [2,28], it may have reduced the statistical power and stability of the hierarchical regression model. Consequently, the present findings should be interpreted with caution and confirmed in larger independent cohorts using external validation and alternative modeling approaches [26,28,29]. At the same time, restricting the analyses to male athletes reduced biological variability associated with sex differences, thereby providing a more homogeneous evaluation of the physiological determinants of sprint kayak performance. Nevertheless, the findings cannot be generalized to female sprint kayakers and should be confirmed in future studies including adequately powered female cohorts. Third, although kayak ergometers provide a highly standardized and reproducible environment for physiological testing, they cannot fully replicate the complex demands of on-water sprint kayaking. In particular, ergometer testing does not reproduce hydrodynamic resistance acting on the kayak and paddle, boat stability, acceleration characteristics, or the continuous adjustments in stroke mechanics required during on-water paddling. These biomechanical differences have been shown to alter muscle activation patterns, force application, and paddling kinematics compared with on-water kayaking. Consequently, although the present findings provide valuable insight into the physiological determinants of laboratory-based sprint kayak performance, caution is warranted when extrapolating these results directly to competitive on-water racing, where biomechanical, technical, tactical, and environmental factors interact to determine performance [31,37,40,41,42,43,44]. Finally, sprint kayak performance is a multifactorial outcome. The present study focused on selected physiological variables; whereas, other potentially important determinants—including anaerobic capacity, neuromuscular characteristics, stroke mechanics, technical efficiency, pacing strategy, psychological factors, and environmental influences—were not evaluated and may also contribute substantially to performance variability [1,2,3,4,5,40,41]. Therefore, the present findings should be interpreted as reflecting the contribution of the physiological variables examined rather than the complete spectrum of determinants of sprint kayak performance. Future studies should integrate physiological, biomechanical, neuromuscular, technical, and on-water performance assessments to provide a more comprehensive understanding of sprint kayaking performance.

## 5. Conclusions

In conclusion, the present study demonstrated that power output at maximal oxygen uptake (POVO_2_max) is the strongest independent aerobic physiological predictor of 500 m sprint kayak performance in national-level male athletes. After accounting for training-group allocation using hierarchical multiple regression, the inclusion of POVO_2_max markedly improved the prediction of sprint performance; whereas, maximal oxygen uptake (VO_2_max) and post-exercise blood lactate concentration did not provide additional independent predictive value. These findings indicate that the ability to generate external mechanical power at maximal aerobic capacity is more closely associated with sprint kayak performance than traditional physiological indices alone. From a practical perspective, the findings support the incorporation of sport-specific power assessments, particularly POVO_2_max, into athlete monitoring and performance evaluation. Routine assessment of POVO_2_max may provide a useful tool for monitoring physiological adaptations and supporting training prescription in competitive sprint kayaking. Future studies involving larger independent cohorts, female athletes, and external validation are warranted to confirm these findings and further refine physiological prediction models for sprint kayak performance.

## Figures and Tables

**Table 1:** Descriptive statistics of post-intervention physiological variables and 500 m sprint kayak performance in male national-level athletes (*N* = 24).

Variable	Unit	Mean ± SD	Min-Max
Age	Yrs	18.6 ± 4.21	16–28
Height	Cm	176.09 ± 8.76	158.0–191.0
Body mass	Kgr	73.09 ± 11.03	56.0–96.0
Body fat	% fat	20.16 ± 4.63	12.6–29.6
VO_2_max	mL·kg^−1^·min^−1^	45.24 ± 7.84	35.2–62.0
POVO_2_max	W	102.80 ± 25.88	70.0–175.0
POVT_2_	W	80.83 ± 19.22	50.0–120.0
PO_75_%VO_2_max	W	76.79 ± 19.83	50.0–131.3
Blood lactate	mmol·L^−1^	10.38 ± 2.75	5.9–16.1
HRmax	beats·min^−1^	196.79 ± 6.52	184–210
500 m performance time	S	127.21 ± 6.07	116.4–143.6

Note: Values are presented as mean ± standard deviation (SD) for male participants following completion of the training intervention (POST). VO_2_max = maximal oxygen uptake; POVO_2_max = power output at maximal oxygen uptake; POVT_2_ = power output at the second ventilatory threshold; PO_75_%VO_2_max = Power output at 75% VO_2_max; HRmax = maximal heart rate.

**Table 2:** Pearson product–moment correlation coefficients between post-intervention physiological variables and 500 m sprint performance time in male national-level sprint kayakers (*N* = 24).

Variable	R	*p*	Magnitude
POVO_2_max (W)	−0.71	<0.001	Very large
POVT_2_ (W)	−0.54	0.006	Large
PO_75_%VO_2_max (W)	−0.69	<0.001	Large
VO_2_max (mL·kg^−1^·min^−1^)	−0.36	0.083	Moderate
HRmax (beats·min^−1^)	0.22	0.291	Small
Blood lactate (mmol·L^−1^)	−0.15	0.487	Small

Note. Negative correlation coefficients indicate that higher physiological values were associated with shorter (faster) 500 m sprint performance times. Correlation magnitudes were interpreted as trivial (<0.10), small (0.10–0.29), moderate (0.30–0.49), large (0.50–0.69), very large (0.70–0.89), and nearly perfect (≥0.90) according to Hopkins et al. [25]. Abbreviations. VO_2_max, maximal oxygen uptake; POVO_2_max, power output at maximal oxygen uptake; POVT_2_, power output at the second ventilatory threshold; Power output at 75% VO_2_max; HRmax, maximal heart rate.

**Table 3:** Hierarchical multiple regression analysis predicting 500 m sprint kayak performance time (*N* = 24).

Model	Predictors	R^2^	Adjusted R^2^	ΔR^2^	*p* (ΔR^2^)
1	Group	0.082	−0.055	–	0.648
2	Group + POVO_2_max	0.708	0.646	0.626	<0.001
3	Group + POVO_2_max + VO_2_max	0.720	0.633	0.012	0.363
4	Group + POVO_2_max + VO_2_max + La	0.724	0.626	0.004	0.855

Note. R^2^ = coefficient of determination; Adjusted R^2^ = adjusted coefficient of determination; ΔR^2^ = increase in explained variance compared with the previous model. Statistical significance of ΔR^2^ was evaluated using the F-change test. Group was entered in Model 1 to account for potential differences related to the original intervention allocation. Physiological variables were subsequently entered hierarchically to determine their incremental contribution to the prediction of 500 m sprint kayak performance time.

**Table 4:** Final hierarchical multiple regression model predicting 500 m sprint kayak performance in male athletes (*N* = 24).

Predictor	B	SE	95% CI	Β	*p*
Group: CON vs. COMB	−8.67	3.19	−15.41 to −1.94	−0.63	0.015
Group: HIIT vs. COMB	−4.53	2.37	−9.53 to 0.47	−0.33	0.073
Group: SIT vs. COMB	−1.65	2.84	−7.63 to 4.34	−0.12	0.569
POVO_2_max	−0.21	0.04	−0.29 to −0.12	−0.88	<0.001
VO_2_max	−0.12	0.13	−0.40 to 0.15	−0.16	0.363
La^+^	0.08	0.42	−0.81 to 0.97	0.04	0.855

## Data Availability

The data presented in this study are available on request from the corresponding author due to privacy and ethical restrictions.

## References

[1] Bassett D.R., Howley E.T. (2000). Limiting factors for maximum oxygen uptake and determinants of endurance performance. Med. Sci. Sports Exerc..

[2] Bishop D. (2000). Physiological predictors of flat-water kayak performance in women. Eur. J. Appl. Physiol..

[3] Michael J.S., Rooney K.B., Smith R.M. (2008). The metabolic demands of kayaking: A review. J. Sports Sci. Med..

[4] Bertozzi F., Porcelli S., Marzorati M., Pilotto A.M., Galli M., Sforza C., Zago M. (2022). Whole-body kinematics during a simulated sprint in flat-water kayakers. Eur. J. Sport Sci..

[5] Bishop D., Bonetti D., Dawson B. (2002). The influence of pacing strategy on VO2 and performance during kayaking. Med. Sci. Sports Exerc..

[6] Coyle E.F. (1995). Integration of physiological factors determining endurance performance ability. Exerc. Sport Sci. Rev..

[7] Hazell T.J., MacPherson R.E.K., Gravelle B.M.R., Lemon P.W.R. (2010). 10 or 30-s sprint interval training bouts enhance both aerobic and anaerobic performance. Eur. J. Appl. Physiol..

[8] Jones A.M., Carter H. (2000). The effect of endurance training on parameters of aerobic fitness. Sports Med..

[9] Joyner M.J., Coyle E.F. (2008). Endurance exercise performance: The physiology of champions. J. Physiol..

[10] Faude O., Kindermann W., Meyer T. (2009). Lactate threshold concepts: How valid are they?. Sports Med..

[11] Midgley A.W., McNaughton L.R., Jones A.M. (2007). Training to enhance the physiological determinants of long-distance running performance. Sports Med..

[12] MacInnis M.J., Gibala M.J. (2017). Physiological adaptations to interval training and the role of exercise intensity. J. Physiol..

[13] Wasserman K., Hansen J.E., Sue D.Y., Stringer W.W., Sietsema K.E. (2011). Principles of Exercise Testing and Interpretation.

[14] García-Pallarés J., Sánchez-Medina L., Carrasco L., Díaz A., Izquierdo M. (2009). Endurance and neuromuscular changes in world-class level kayakers during a periodized training cycle. Eur. J. Appl. Physiol..

[15] García-Pallarés J., Sánchez-Medina L., Pérez C.E., Izquierdo-Gabarren M., Izquierdo M. (2010). Physiological effects of tapering and detraining in world-class kayakers. Med. Sci. Sports Exerc..

[16] García-Pallarés J., Izquierdo M. (2011). Strategies to optimize concurrent training of strength and aerobic fitness for rowing and canoeing. Sports Med..

[17] Billat L.V. (2001). Interval training for performance: A scientific and empirical practice. Sports Med..

[18] Buchheit M., Laursen P.B. (2013). High-intensity interval training: Solutions to the programming puzzle: Part I. Sports Med..

[19] Buchheit M., Laursen P.B. (2013). High-intensity interval training: Solutions to the programming puzzle: Part II. Sports Med..

[20] Gibala M.J., Little J.P., MacDonald M.J., Hawley J.A. (2012). Physiological adaptations to low-volume high-intensity interval training. J. Physiol..

[21] Gist N.H., Fedewa M.V., Dishman R.K., Cureton K.J. (2014). Sprint interval training effects on aerobic capacity: A systematic review and meta-analysis. Sports Med..

[22] Laursen P.B., Jenkins D.G. (2002). The scientific basis for high-intensity interval training. Sports Med..

[23] Rosenblat M.A., Perrotta A.S., Thomas S.G. (2020). Effect of high-intensity interval training versus sprint interval training on time-trial performance: A systematic review and meta-analysis. Sports Med..

[24] Weston M., Taylor K.L., Batterham A.M., Hopkins W.G. (2014). Effects of low-volume high-intensity interval training on fitness in adults: A meta-analysis of controlled and non-controlled trials. Sports Med..

[25] Alvero-Cruz J.R., Carnero E.A., García M.A., Alacid F., Correas-Gómez L., Rosemann T., Nikolaidis P.T., Knechtle B. (2020). Predictive Performance Models in Long-Distance Runners: A Systematic Review. Int. J. Environ. Res. Public Health.

[26] Lee Y.S., Dingley A., Lum D., Tan F., Fernandes J.F.T. (2025). Physiological and Physical Determinants of Flat-Water Kayaking. Muscles.

[27] Wang X., Zhao L. (2023). Comparative Analysis of Cardiorespiratory Fitness, Bio-Motor Abilities, and Body Composition Indicators among Sprint Kayakers of Different Age Groups and Expertise Levels. Front. Physiol..

[28] Gäbler M., Prieske O., Hortobágyi T., Granacher U. (2021). Measures of Physical Fitness Improve Prediction of Kayak and Canoe Sprint Performance in Young Kayakers and Canoeists. Sports.

[29] Álvarez-Yates T., García-García O. (2021). Determinants of flatwater canoeing and kayaking performance: A systematic review. Med. Dello Sport.

[30] World Medical Association (2013). Declaration of Helsinki: Ethical Principles for Medical Research Involving Human Subjects. JAMA.

[31] Van Someren K.A., Oliver J.E. (2002). The efficacy of ergometer training in kayaking. Int. J. Sports Med..

[32] Midgley A.W., McNaughton L.R., Wilkinson M. (2006). Criteria for determination of maximal oxygen uptake. Sports Med..

[33] Beaver W.L., Wasserman K., Whipp B.J. (1986). A new method for detecting anaerobic threshold by gas exchange. J. Appl. Physiol..

[34] Buchheit M. (2014). Monitoring training status with HR measures: Do all roads lead to Rome?. Front. Physiol..

[35] Plews D.J., Laursen P.B., Stanley J., Kilding A.E., Buchheit M. (2013). Training Adaptation and Heart Rate Variability in Elite En-durance Athletes: Opening the Door to Effective Monitoring. Sports Med..

[36] Papandreou A., Philippou A., Zacharogiannis E., Maridaki M. (2020). Physiological Adaptations to High-Intensity Interval and Continuous Training in Kayak Athletes. J. Strength Cond. Res..

[37] Hopkins W.G. A Scale of Magnitudes for Effect Statistics. A New View of Statistics 2002. http://www.sportsci.org/resource/stats/effectmag.html.

[38] Field A. (2018). 39. Discovering Statistics Using IBM SPSS Statistics.

[39] Fleming N., Donne B., Fletcher D., Mahony N. (2012). A Biomechanical Assessment of Ergometer Task Specificity in Elite Flatwater Kayakers. J. Sports Sci. Med..

[40] Seifert L., Boulesteix L., Chollet D. (2008). Effect of fatigue on kayaking technique. J. Sports Sci..

[41] Skorski S., Abbiss C.R. (2017). The Manipulation of Pace within Endurance Sport. Front. Physiol..

[42] Papandreou A., Tzanis G., Moustogiannis A., Zevolis E., Zacharogiannis E., Maridaki M., Nanas S., Koutsilieris M., Philippou A. (2025). Molecular, Systemic, and Physiological Adaptations to High-Intensity Interval Training in Flatwater Kayak Athletes. Sports.

[43] McDonnell L.K., Hume P.A., Nolte V. (2012). An Observational Model for Biomechanical Assessment of Sprint Kayaking Technique. Sports Biomech..

[44] Gomes B.B., Ramos N.V., Conceição F.A.V., Sanders R.H., Vaz M.A.P., Vilas-Boas J.P. (2015). Paddling Force Profiles at Different Stroke Rates in Elite Sprint Kayaking. J. Appl. Biomech..

